# Polymer-Based Wound Dressing Materials Loaded with Bioactive Agents: Potential Materials for the Treatment of Diabetic Wounds

**DOI:** 10.3390/polym14040724

**Published:** 2022-02-14

**Authors:** Sibusiso Alven, Sijongesonke Peter, Zintle Mbese, Blessing A. Aderibigbe

**Affiliations:** Department of Chemistry, University of Fort Hare, Alice 5700, South Africa; 201414787@ufh.ac.za (S.P.); 201208394@ufh.ac.za (Z.M.); baderibigbe@ufh.ac.za (B.A.A.)

**Keywords:** diabetic wounds, polymers, wound dressings, bioactive agents, and diabetic foot ulcer

## Abstract

Diabetic wounds are severe injuries that are common in patients that suffer from diabetes. Most of the presently employed wound dressing scaffolds are inappropriate for treating diabetic wounds. Improper treatment of diabetic wounds usually results in amputations. The shortcomings that are related to the currently used wound dressings include poor antimicrobial properties, inability to provide moisture, weak mechanical features, poor biodegradability, and biocompatibility, etc. To overcome the poor mechanical properties, polymer-based wound dressings have been designed from the combination of biopolymers (natural polymers) (e.g., chitosan, alginate, cellulose, chitin, gelatin, etc.) and synthetic polymers (e.g., poly (vinyl alcohol), poly (lactic-co-glycolic acid), polylactide, poly-glycolic acid, polyurethanes, etc.) to produce effective hybrid scaffolds for wound management. The loading of bioactive agents or drugs into polymer-based wound dressings can result in improved therapeutic outcomes such as good antibacterial or antioxidant activity when used in the treatment of diabetic wounds. Based on the outstanding performance of polymer-based wound dressings on diabetic wounds in the pre-clinical experiments, the in vivo and in vitro therapeutic results of the wound dressing materials on the diabetic wound are hereby reviewed.

## 1. Introduction

Diabetes is a chronic condition with a high incidence of mortality and numerous complications that include diabetic foot ulcers (DFU) [1]. In 2013, it was reported that approximately 366 million individuals suffered from diabetes worldwide and in 2019, 1.5 million deaths were caused by diabetes [2]. Diabetes is a medical condition due to the inability of the pancreas to produce sufficient insulin or the inability of the body to effectively use the insulin produced [3]. Diabetic patients usually suffer from chronic injuries such as DFU and diabetic ulcers/leg ulcers. These wounds display features of a prolonged wound healing process and result in hospitalization and limb amputations [4]. About 50–70% of limb amputations are caused by diabetic injuries, and it has been reported globally that one leg is amputated every 30 s because of diabetic wounds [5,6]. Various factors result in delayed healing of diabetic wounds that are often taken into consideration by biomedical researchers, such as deformity, peripheral neuropathy, hanging on microcirculation function, macrovascular disease, peripheral arterial disease, cardiovascular events, kidney disease, and the disruption to growth factors (GFs) activity and expression [7,8,9].

Wound dressing materials based on polymers have attracted much attention in the management of chronic wounds, especially diabetic injuries. These dressings demonstrate several interesting properties that can be beneficial for the management of chronic injuries. The properties of ideal polymeric dressings include high porosity and swelling ability, adequate water vapour transmission rate (WVTR), ability to offer moisture and warm environment to accelerate the wound healing process, gaseous permeation, excellent antimicrobial properties, excellent mechanical performance, and capability to deliver bioactive agents [10,11,12]. Various polymers can be utilized for the formulation of ideal wound dressing materials. They are categorized as biopolymers and synthetic polymers. Examples of biopolymers (natural polymers) include alginate, dextran, hyaluronic acid (HA), chitosan, cellulose, gelatin, chitin, etc. [13]. These polymers present excellent biomedical properties such as good biocompatibility, non-immunogenicity, non-toxicity, hemostatic effects, excellent biodegradability, antibacterial features, and wound healing properties [14]. However, natural polymers also suffer from poor mechanical properties. 

Natural-based polymeric wound dressing materials are usually cross-linked with synthetic polymers to enhance their mechanical performance [14]. The synthetic polymers that can be cross-linked with natural polymers for wound dressing formulations include poly(vinyl alcohol) (PVA), poly(lactic-co-glycolic acid) (PLGA), polylactide (PLA), polyglycolic acid (PGA), polyurethanes (PUs), poly(ethylene oxide) (PEO)/poly(ethylene glycol) (PEG), poly(hydroxyethyl methacrylate) (PHEMA), and poly(vinyl pyrrolidone) (PVP) [15]. The cross-linked dressings can also exhibit poor biological activities and weak wound healing properties, making them inappropriate for managing diabetic wounds. The encapsulation of bioactive agents in these polymer-based dressings has been reported to be a promising approach for wound care, precisely chronic wounds [16]. The bioactive agents used in wound healing applications include antibiotics, GFs, stem cells, plant extracts, antioxidants, anti-inflammatory drugs (e.g., curcumin, etc.), and vitamins. Some polymeric wound dressings loaded with the above-mentioned bioactive agents are hydrogels, foams, membranes, films, nanofibers, transdermal patches, etc. [17]. This review article discusses the in vivo and in vitro therapeutic results of polymer-based wound dressing scaffolds encapsulated with various bioactive agents for the treatment of diabetic wounds. 

## 2. Classification of Wounds and Phases of Healing Process in Diabetic Wounds

Wounds are generally defined as damaged on the epidermal or even dermal layer of the skin. The usual causes of injuries include corrosive chemicals, electricity, sharp objects, gunshots, heat and fires, and diseases such as diabetes mellitus, etc. [18]. Wounds are mainly categorized based on their wound healing time as acute or chronic wounds. The acute wounds usually recover within the timeframe of 4–12 weeks depending on their depth, size, and intensity on the epidermis or dermis lining of the skin [19]. However, improper treatment of acute injuries can result in them becoming chronic wounds. Chronic wounds do not heal through the normal wound healing phases [20]. The factor contributing to chronic wounds includes age, obesity, prolonged bed rest, malnutrition, smoking, diseases, and microbial invasion. Examples of chronic wounds are burns, leg ulcers, and diabetic wounds [20]. 

**Figure 1:**
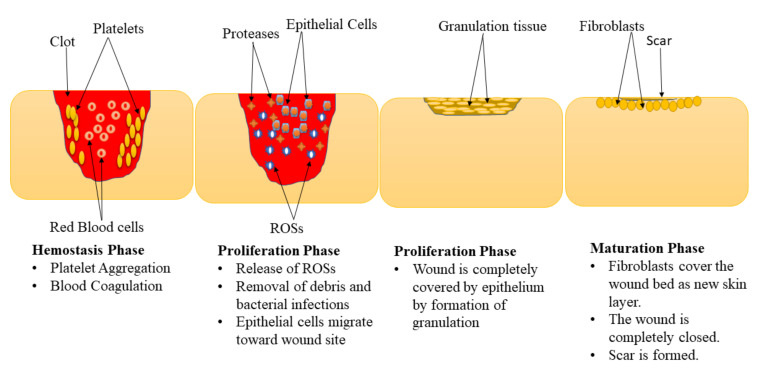
Phases of the wound healing process.

Wound healing is a complex physiological mechanism that involves the interaction of various GFs, cells, proteinases, and extracellular matrix (ECM) constituents for the restoration of anatomic integrity with analogous function [21,22,23]. This process is comprised of 4 phases: hemostasis, inflammation, proliferation, and maturation (remodeling) phase (Figure 1) [24,25]. These phases are impaired in diabetic wounds leading retarded healing process, especially the inflammation and proliferation phase [26,27]. The hemostasis stage happens instantly after the wound, promoting blood coagulation and exudate to stop the bleeding [28]. The inflammation phase normally takes place simultaneously with the hemostasis phases. In this phase, debris is removed, protecting the wound from microbial invasion. The macrophages release numerous cytokines and GFs that recruit endothelial cells, keratinocytes, and fibroblasts to promote angiogenesis [29]. Furthermore, the epithelial cells invade towards the injury site to substitute dead cells. 

In the proliferation phase, the epithelium covers the wound with the development of granulation tissues [30]. Diabetic wounds remain in the inflammatory phase resulting in the inhibition of the formation of matured granulation tissue and reduces injury tensile strength. This is caused by vascular damage leading to ischemia. The final phase of the wound healing process is the maturation phase also called the remodelling stage. In the maturation phase, the injury is fully closed. The fibroblasts completely cover the surface of the injury resulting in tissue remodelling and the formation of a new skin epidermal layer. This process leads to wound closure that is caused by the differentiation of fibroblast cells into contractile myofibroblasts [31]. 

## 3. Factors That Impede the Healing of Diabetic Wounds

Various factors usually impede the healing process of diabetic wounds and related wounds. However, the main factors that impede diabetic wound healing include vasculopathy, neuropathy, infections, immune system deficiency, and interrupted growth factor activity, cellular dysfunction, and poor oxygenation [29].

### 3.1. Vasculopathy

Type 1 diabetes is associated with macrovascular diseases, and the distal arteries are unable to deliver nutrients and oxygen to the injury site resulting in the delayed wound healing process. Diabetes changes the circulation of distal vessels originating from the pedal and femoral arteries together with metatarsal arteries [32]. These microcirculatory shortages happen in the primary phases of diabetes. Consequently, arteriolar deficiencies, thickening of the basement membrane, and decline in the capillary size happen. The increase in the width of the basement membrane limits physiological exchanges and result in decreased hyperemia at the highest level, transform leucocytes migration, and asymmetrical autoregulatory capability [33]. In addition to these microcirculatory shortages, endothelial cell dysfunction also happens that may result in reduced crucial physiological function such as that from an enzyme called nitric oxide synthetase. Due to endothelial cell dysfunction, arterioles and arteries may not optimally dilate causing suboptimal wound healing and irregular blood flow [34].

### 3.2. Neuropathy

Motor, autonomic, and sensory fibres are disrupted in patients with diabetes, and sensory deficiencies result in a failure to sense outward stimuli such as heat, pressure, and injuries. Consequently, the wound recovery process can be delayed in diabetes patients [6]. The absence of pain together with the abnormal vasodilator autoregulation also lead to complications that further delay the wound healing process. Motor fibre defects together with the aforementioned complications result in unwanted physical stress and deteriorating of injuries. Therefore, neuropathy can cause the progression of bacterial burden and infection in tissue [6]. 

### 3.3. Infections

Infections are among the significant reasons for morbidity in diabetic patients with outcomes such as delayed wound healing, hospitalization, and amputation. Infections can happen very rapidly in diabetic injuries. Conditions such as abscesses, osteomyelitis, and cellulitis require proper care [35]. Persistent hypoxia at the injury bed is detrimental to wound healing and may lead to reperfusion of the wound by creating oxygen radicals [36]. 

### 3.4. Immune System Deficiency

The immune system in the body of a patient with diabetes is also usually affected. The several immune fighter cells that are responsible for healing injuries and their ability to work is severely reduced because of diabetes [37]. The affected function of the immune system results in a delayed wound healing process and therefore, the wound is prone to the risk of infection. If the immune system is not functioning properly, it becomes challenging for the injury to fight off bacterial infections. High levels of blood sugar can also stop immune cells from combating the invading bacteria. Untreated infections can lead to complications such as sepsis or gangrene [37].

### 3.5. Interrupted Growth Factor Activity

Growth factors are biological polypeptides that play a critical role almost in all phases of the wound healing process. These polypeptides stimulate the initial inflammation phase that occurs during the granulation phase of tissue development [5]. Examples of growth factors that are significantly involved in the wound healing mechanism include transforming growth factor β (TGF-β), fibroblast growth factor (FGF), epidermal growth factor (EGF), basic fibroblast growth factor (bFGF), keratinocyte growth factor (KGF), vascular endothelial growth factor (VEGF), and platelet-derived growth factor (PDGF). These factors are prominently decreased in patients with diabetes and then result in a delayed wound healing process [5]. The decreased levels of TGF-β1 increases the recruitment of activated inflammatory cells leading to a delayed inflammatory phase to the proliferation phase in the diabetic wound healing process [38].

### 3.6. Cellular Dysfunction

Cellular dysfunction of various skin cell types such as keratinocytes, fibroblasts, endothelial cells, and macrophages can result in a delayed wound healing process of diabetic injuries. The mechanism of how cellular dysfunction delays diabetic wound healing is not well-known. Some studies conducted by Liang et al., revealed that the presence of microRNAs (miR-145-5p, miR-34a-5p, and miR-21-5p) in DFU fibroblasts caused impaired multiple cellular functions, leading to an overall hindering of wound recovery in DFUs. The wound dressings can be loaded with bioactive agents that possess the efficacy to inhibit the activity of those micro-RNA [39].

### 3.7. Poor Oxygenation

Oxygen is one of the vital factors almost in all wound healing mechanisms due to its ability to demonstrate important roles for cell metabolism, particularly in the production of energy via the use of adenosine triphosphate (ATP). It stimulates wound closure, enhances migration, differentiation, and re-epithelization of keratinocytes, induces angiogenesis, prevents bacterial invasion to the wounds, and increases fibroblast proliferation and collagen formation [40]. Oxygen is involved in all the phases of the wound healing process (the inflammation, proliferation, and remodelling phase). The poor oxygenation that usually occurs in diabetic wounds severely affect the wound healing process by causing the wound to remain in the inflammatory stage for a prolonged period [40].

## 4. Classification of Wound Dressings 

**Figure 2:**
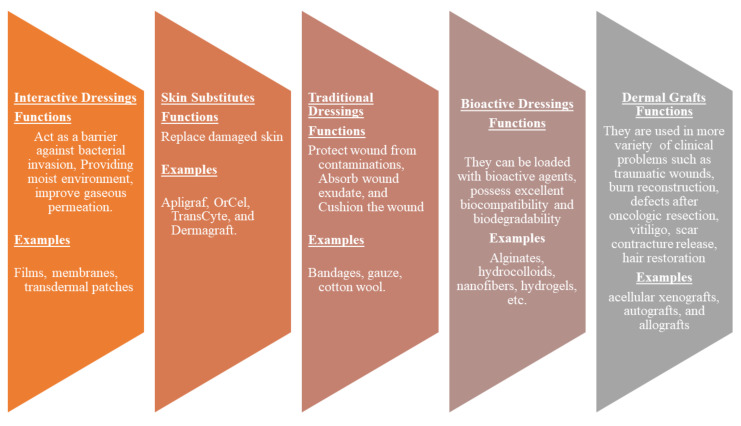
Classification of wound dressings.

Wound dressings play a vital role in the treatment of various injuries. The primary function of wound dressings is their ability to protect wounds from bacterial invasion and accelerate the wound healing process [41]. However, some of the presently utilized wound dressings display delayed healing processes, poor gaseous permeation, inability to provide moisture, induce allergic reactions, etc. [42]. There is an urgent need to develop effective wound dressing materials. Wound dressing can be classified into four well-known groups: traditional/passive, interactive materials, skin substitutes, and bioactive dressings (Figure 2). Traditional dressings primarily keep the injury from foreign substances or contamination, stop bleeding, cushion the injury, and absorb wound exudate. The examples include wool dressing, plaster, gauze, and bandages [43]. Some of these dressings suffer from shortcomings such as leaking wound exudate resulting in bacterial infections and cause harm to the skin during removal [44]. 

Interactive dressings such as composites, films, gels, foams, sprays possess the ability to accelerate wound healing by offering a moist environment, displaying good water transmission, and enhancing re-epithelialization and granulation [45,46]. These wound dressings can also be loaded with bioactive agents. Skin substitutes such as Apligraf, OrCel, and TransCyte are composed of tissue-engineered structures, typically arising from cell co-culture or cell-seeded scaffold materials, and they are effective in skin regeneration. However, they can cause wound infections, transmit diseases, can be rejected by the body, are expensive, and have limited shelf life [45]. Dermal grafts are one of the most necessary materials in the field of dermatology and plastic surgery. Examples of dermal grafts are acellular xenografts, autografts, and allografts. These materials are used in traumatic wounds, burn reconstruction, defects after oncologic resection, vitiligo, scar contracture release, hair restoration, and congenital skin deficiencies. Nevertheless, they are not suitable for the management of complex injuries (i.e., conditions with exposed bones and deep spaces) [47]. Bioactive dressings such as hydrocolloids, sponges, wafers, foams, nanofibers, hydrogels, collagens, films are biodegradable, biocompatible, and can act as drug delivery systems for therapeutic agents such as nanoparticles, GFs, vitamins, antibiotics with improved wound healing process [48,49]. Although nanofibers and hydrogels are other types of wound dressings. They can be loaded with drugs and display controlled drug delivery of bioactive agents, making them bioactive wound dressings.

## 5. Polymer-Based Dressings Loaded with Bioactive Agents for Diabetic Wound Management

### 5.1. Nanofibers

**Figure 3:**
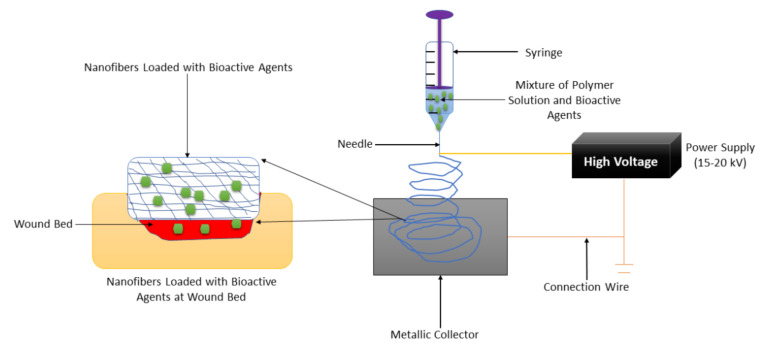
Nanofiber formulation by the electrospinning technique.

Nanofiber-based wound dressings have a mean diameter size of less than 1 micrometer [50]. They are easily removed from the injury after their application. Among the preparation methods that are employed to fabricate nanofibers, electrospinning is the most employed one because of its several advantages such as adjustment of mechanical properties of nanofibers, control nanofiber porosity, cost-efficiency, simplicity, and versatility [51,52,53]. The formulation of nanofibers using the electrospinning technique is shown in Figure 3. Nanofibers present many advantages, including a small diameter, high porosity, narrow diameter distribution, gas permeation, and high-specific surface to area ratio [54]. These wound dressings have been used for drug delivery, especially for the management of chronic injuries [55]. They employ the accessories or excipients to transport the drugs to the wound with low toxicity and high efficiency. Nanofibrous wound dressings display related diameters with the extracellular matrix (ECM), making them suitable for improving wound healing and supporting cell proliferation and adhesion [56]. Many research reports have discussed the therapeutic outcomes of nanofibers in diabetic wound management. Most of the nanofibers were formulated from poly (α-esters) (PLA, PGA, and PLGA), chitosan, gelatin, chitosan, HA, and alginate. 

Cam et al., fabricated bacterial cellulose-gelatin nanofibers co-loaded with glibenclamide and metformin for diabetic wound treatment [57]. The scanning electron microscope (SEM) results of drug-encapsulated nanofibers exhibited bead-less and uniform structure (with a fiber diameter of 0.22 μm), mimicking that one of ECM. The cytotoxicity studies in vitro displayed high cell viability of mouse fibroblasts (L929) when they were incubated with plain nanofibers and glibenclamide/metformin-loaded nanofibers for 48 h, indicating non-toxicity and good biocompatibility of nanofibers [57]. The encapsulation efficiencies of glibenclamide and metformin in hybrid nanofibers were ~78% and ~80%, respectively. The in vivo wound healing studies performed on drug-loaded fibers using type -1 diabetic Wistar rats displayed superior wound healing on full-thickness wounds than the pristine nanofibers, revealing that glibenclamide and metformin significantly accelerated diabetic wound healing [57]. The co-loading of bioactive agents promoted an effective wound healing process. Choi et al., formulated PEG-PCL hybrid nanofibers encapsulated with human epidermal growth factor (EGF) using the electrospinning technique for the treatment of diabetic ulcers. The in vivo wound closure studies utilizing full-thickness injuries on streptozotocin (STZ)-induced diabetic mice demonstrated that the injury treated with EGF-encapsulated nanofibers was superior healed on day 7 than those dressed with plain nanofibers or EGF alone [58]. The EGF-loaded significantly increased the rate of wound healing process resulting in complete wound closure in 7 days than the usual (14 or 15 days). 

Cui et al., fabricated doxycycline-loaded polylactide-based nanofibers for chronic wound management [59]. The contact angle measurement experiments demonstrated that the high content of doxycycline importantly enhances the hydrophilicity of nanofibers. The mechanical characterizations of drug-loaded nanofibers showed the tensile strength of 4.74 ± 0.64 MPa while water vapor transmission rate (WVTR) studies exhibited WVTR of 0.4041 ± 0.0001 g/(cm^2^·24 h). The in vitro drug release experiments underphysiological conditions (temperature 37° and pH 7.4) showed that the doxycycline was rapidly released from the nanofibers followed by a sustained drug release for 2 weeks at high drug content. The antibacterial analysis in vitro of doxycycline-encapsulated nanofibers demonstrated a high zone of inhibition against *Escherichia coli* (*E. coli)* and *Staphyloccocus aureus* (*S. aureus*) while pristine nanofiber didn’t display any antibacterial efficacy. The wound healing studies in vivo exhibited that the contraction of full-thickness diabetic injuries was much faster when dressed with drug-loaded nanofibers than those treated with pristine nanofibers and free drugs [59]. The doxycycline-loaded nanofibers demonstrated excellent antibacterial efficacy, making these nanofiber scaffolds potential candidates for the treatment of infected diabetic wounds. Ranjbar-Mohammadi et al., developed polymer-based nanofibers from PCL and gum tragacanth incorporated with curcumin for diabetic wound care. The SEM micrographs of nanofibers exhibited bead-less morphology. The in vivo wound closure experiments utilizing diabetic Sprauge Dawrely (SD) rats exhibited that the areas of the full thickness wounds dressed with curcumin-loaded hybrid nanofibers completely closed on day 15 while those treated with control samples decreased to only 20.96 ± 1.35%, revealing that curcumin-loaded hybrid nanofibrous scaffolds are potential systems for diabetic wound management because of interesting wound healing effects of curcumin [60].

Almasian et al., reported PU-carboxymethylcellulose nanofibers loaded with *Malva sylvestris* plant extract for diabetic wound treatment. The diabetic wound closure studies demonstrated the average healing rate for gauze bandages, plain hybrid nanofibers, and plant extract-loaded nanofiber dressings of about 32.1 ± 0.2%, 51.4 ± 0.4%, and 95.11 ± 0.2%, respectively on day 14 [61]. The nanofiber wound dressings loaded with *Malva sylvestris* were superior for the management of diabetic wounds when compared to the traditional methods (e.g., gauze) due to the presence of *Malva sylvestris*. Grip et al., fabricated hydroxypropyl methylcellulose-polyethylene oxide (PEO) nanofibers loaded with β-glucan for diabetic wound care [58]. The FTIR data demonstrated that the nanofibers were successfully fabricated. The in vitro cytocompatibility utilizing MTT assay exhibited high cell viability of keratinocytes when were cultured with beta-glucan-loaded nanofibers, indicating that these nanofibers can not cause any toxicity in the proliferation phase. The in vivo studies using diabetic mice exhibited that the beta-glucan-nanofibers significantly improved wound contraction in comparison to the pristine hybrid nanofibers [62]. Good cytocompatibility of hydroxypropyl methylcellulose-polyethylene oxide (PEO) nanofibers have the potential to significantly lead to high cell proliferation of skin cells which is suitable for the accelerated wound healing process of diabetic injuries. The poly-N-acetylglucosamine nanofibers reported by Chen et al., were encapsulated with polydeoxyribonucleotide. The nanofibers demonstrated promoted cell proliferation of fibroblast and new vessel development and superior wound recovery process on a diabetic skin ulcer mouse model [63].

Kanji et al., synthesized polyethersulfone nanofibers loaded with human umbilical cord blood-derived CD34+ cells (henceforth CD34+ cells) for diabetic wound management. The in vivo studies utilizing STZ induced diabetic mice showed that the injuries treated with CD34+ cell-loaded nanofibers were completely healed on day 11 post-surgery, while those dressed with plain nanofiber were still open on day 15 [64]. The CD34+ are major bioactive agents that contributed to the accelerated wound healing mechanism of the diabetic wound in vivo. Pinzón-García reported bixin-loaded PCL nanofibers for diabetic wound healing applications. The XRD and FTIR data showed the successful preparation of Bixin-incorporated nanofibers. The drug release experiments in vitro showed an initial rapid drug release of bixin from the nanofibers followed by a constant release manner. The in vivo wound closure outcomes on the excisional diabetic wound model in rats demonstrated that the low concentration of Bixin released from encapsulated Bix-PCL nanofibers maintains the therapeutic efficacy of Bixin and is effective in accelerating the wound healing process when compared with pristine nanofibers [65]. The sustained release of bixin from PCL nanofibers resulted in improved wound healing effects of the nanofibers. Lee et al., fabricated PLGA nanofibers encapsulated with PDGF, vancomycin, and gentamicin for diabetic-infected wound care. The drug release kinetics displayed sustained release of loaded antibiotics for 21 days. The in vivo experiments displayed a fast healing rate in the early stage of infected diabetic wound healing [66]. The prolonged and sustained drug release of antibiotics from PLGA nanofibers for 21 days significantly resulted in the accelerated rate of the wound healing process.

Zehra et al., formulated nanofibers that are based on PCL incorporated with sodium percarbonate for diabetic wound management. The FTIR and EDS confirmed the physicochemical properties of nanofibers. The in vivo wound closure studies using STZ-induced diabetic rats exhibited that the full thickness wounds wrapped with sodium percarbonate-loaded nanofibers had a superior vascularized and compact ECM with entirely covered thick epithelium [67]. The loading of percarbonate into polymeric nanofibers significantly promoted vital wound healing processes suitable to accelerate diabetic wound healing. Shalaby et al., prepared cellulose acetate-based nanofibers loaded with silver (Ag) nanoparticles for the treatment of microbial infected diabetic lesions. The in vitro antibacterial studies displayed that the antibacterial effects of nanofibers increased against *E. coli* and *S. aureus* as the content of Ag nanoparticles increase. The in vivo studies demonstrated that diabetic lesions in mice treated with nanoparticle-loaded nanofiber exhibited significantly accelerated wound reduction compared to those treated with insulin [68]. Ag nanoparticles improved the antimicrobial effects of the nanofibers, making the polymeric nanofibers display rapid wound contraction. Merrel et al., designed PCL-based nanofibers encapsulated with curcumin for the management of diabetic wounds. The in vitro cytotoxicity studies exhibited more than 70% cell viability of Human foreskin fibroblast cells (HFF-1) when incubated with curcumin-loaded nanofibers, revealing good biocompatibility. The in vivo experiments with the curcumin-encapsulated nanofibers exhibited an acceleration of wound closure in an STZ-induced diabetic mice model [69]. The non-toxicity of the scaffolds to the skin cells and the wound healing effects of curcumin in nanofibers induced an improved healing process of the wounds. Lee et al., fabricated electrospun insulin-loaded PLGA nanofibrous materials for diabetic wound recovery. The mechanical analysis of insulin-loaded nanofibers displayed elongation at a break of 164.3 ± 27.2% and tensile strength of approximately 2.87 ± 0.07 MPa, similar to human native skin. The core-shell nanofibrous scaffolds prolong insulin release in vitro and significantly stimulated rat diabetic wound healing [70]. The enhanced mechanical performance and prolonged drug release of the nanofibrous materials significantly led to an improved wound process. 

Ahmed et al., fabricated PVA-chitosan nanofiber mats incorporated with zinc oxide (ZnO) nanoparticles for microbial-infected diabetic wound care [71]. The successful fabrication of nanoparticle-loaded nanofiber mats was demonstrated by FTIR and XRD data. The SEM images of nanofiber mats showed uniform and bead-free morphology mimicking that one of ECM. The in vitro studies displayed the higher zone of inhibitions of ZnO-loaded PVA- chitosan nanofiber mats against *E. coli*, *S. aureus*, *Pseudomonas aeruginosa* (*P. aeruginosa*), and *Bacillus subtilis* (*B. subtilis*) were 20.2 ± 1.0, 15.5 ± 0.8, 21.8 ± 1.5 and 21.5 ± 0.5 mm, respectively than pristine mats which were 14.1 ± 0.8, 13.0 ± 0.7, 15.8 ± 1.0 and 5.4 ± 0.5 mm respectively, indicating that both mats have significant antibacterial potential. The in vivo experiments using subcutaneous wounds in diabetes-induced rabbits demonstrated 90.5 ± 1.7% wound contraction on day 12 for injuries dressed with ZnO-loaded nanofiber mats. In comparison, those treated with control exhibited only 52.3 ± 2.8% wound closure [71]. The high antimicrobial efficacy of ZnO nanoparticles in nanofibrous mats is a factor that led to the rapid wound closure of diabetic injuries. The pioglitazone-loaded PVP-PCL nanofibrous mats reported by Cam et al., demonstrated initially rapid release of drug followed by sustained release manner with the fast diabetic wound healing process in type-1 diabetic mice, and pioglitazone-loaded nanofiber mats did not demonstrate any cytotoxic effect on mouse embryo fibroblast (NIH/3T3) cells [72]. The sustained release of pioglitazone from the hybrid mats and their good cytocompatibility resulted in an accelerated wound healing process. 

Most of the SEM results of drug-loaded nanofibers exhibited a bead-free morphology that mimics ECM, indicating that these nanofibers can provide a suitable environment for cell growth and attachment during the wound recovery process. The combination of two or more polymers for the formulation of nanofibers (especially in the case of natural polymers) result in good mechanical properties that are crucial for diabetic wound management. The nanofibers loaded with bioactive agents demonstrate an accelerated wound healing process in the in vivo experiments when compared with plain nanofibers and controls, suggesting that the loading of bioactive agents plays a significant role in the treatment of diabetic wounds. The encapsulation of two drugs in nanofibers even results in good therapeutic outcomes that may be due to synergistic effects. Furthermore, the drug release profiles exhibited initial rapid release of loaded bioactive agents from the nanofibers followed by sustained drug release, this is an important mechanism that can result in destroying bacterial strains in infected diabetic wounds and further protect the wound with reduced drug resistance. Nevertheless, the content of loaded drugs in nanofibers must be considered because of toxicity concerns. However, the in vitro cytotoxicity experiments nanofibers loaded with bioactive agents have confirmed that these scaffolds possess good biocompatibility and non-toxicity when were incubated with various types of skin cells. These materials are promising candidates that can be employed as effective wound dressings for diabetic wound treatment. However, there is limited diversity of polymers that can be used in the formulation of organic nanofibers through the electrospinning technique. Furthermore, the fabrication of nanofibers with a diameter of less than 10 nm is a problem because such diameter can result in good biological outcomes in diabetic wound management as it mimics ECM.

### 5.2. Films and Membranes 

**Figure 4:**
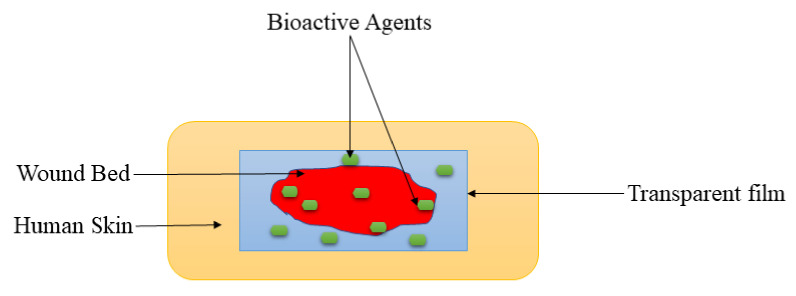
Transparent film at the wound bed.

Films are wound dressing materials that are usually formulated adherent and transparent PU, which permits the permeation of gases such as oxygen, water vapor, and carbon dioxide between the injury and the surrounding [73]. These dressing materials also useful for autolytic removal of dead tissues from the injury. The polymer-based films display excellent mechanical properties, including high elasticity and flexibility, leading to their ability to be altered to any shape of interest, and do not require extra tapping [74]. The transparency of film dressings provides the inspection of the recovery process of the wound without removing the dressing (Figure 4), making them appropriate for wound management of superficial wounds, epithelizing injury with low exudates, and shallow wounds [75]. Tan et al., prepared sodium alginate-based hydrocolloid films incorporated with vicenin-2 for diabetic wound management [76]. The in vivo wound closure studies employing diabetic STZ-induced SD rats showed that the lesions wrapped with vicenin-2-encapsulated films induced faster healing than those dressed with plain films. Also, the histological experiments indicated that pristine film-dressed diabetic wounds exhibited incomplete reepithelialization and poorly developed granulation tissue, while the vicenin-2 film-dressed diabetic rats showed moderate reepithelialization with well-developed granulation tissue after 2 weeks of treatment [76]. The incorporation of vicenin into films significantly promoted the important processes (re-epithelization and granulation) of wound healing. 

Colobatiu et al., reported chitosan-based films encapsulated with alcoholic extracts of various plants such as *Symphytum officinale, Plantago lanceolata, Tagetes patula, Arnica montana, Geum urbanum*, and *Calendula officinalis* for diabetic wound dressing application [77]. These biopolymeric films displayed acceptable appearance, colour, structure, and flexibility as well as a good swelling ability, thus demonstrating a significant capability to prevent wound dehydration. The in vitro cytotoxicity experiments utilizing MTT assay displayed more than 80% cell viability of the Hs27 human fibroblast cells when incubated with bioactive extracts-loaded films, revealing good biocompatibility and non-toxicity. The in vivo experiments on diabetic STZ-induced Wister rats demonstrated that the injuries dressed with the bioactive-loaded films were observed to be almost fully closed (97.47%) on day 14, compared to the plain films that displayed only a 61.07% wound closure. Furthermore, histopathological analysis of chitosan-based films showed an important wound repairing ability, which could stimulate reepithelialization and hasten the wound healing mechanism in diabetic as well as normal wounds [77]. The non-cytotoxic effects of films loaded with alcoholic extracts and other factors resulted in an improved diabetic wound healing process. The chitosan-based films were also formulated by Mizuno et al., that were loaded with fibroblast growth factors. The in vivo wound healing study showed high wound closure of full-thickness wounds on diabetic rats when treated with chitosan films when compared to control [78]. The growth factors play a vital role in wound healing and accelerated the wound healing process. 

Voss et al., fabricated cellulose-PVA films encapsulated with propolis and/or vitamin C for diabetic wound management [79]. The SEM micrographs of films demonstrated homogeneity and the good distribution of cellulose within the PVA matrix. The drug release profiles in vitro displayed that vitamin C was released from films in a controlled manner. The water uptake and swelling analysis using simulated wound fluid showed the films possess high absorptive capability with an equilibrium swelling ratio of more than 200%. Moreover, hybrid films significantly demonstrated a fast swelling rate just before their incubation in wound fluid, making them potential dressings for cell adhesion and accelerated wound healing process. The antimicrobial studies in vitro demonstrated excellent antibacterial activity of dual drug-loaded films against *S. aureus* and *E. coli* compared to single drug-loaded films and plain films. The in vivo studies exhibited that wound healing on diabetic SD rats was significantly accelerated when wounds were treated with dual drug-loaded films [79]. The water uptake and swelling capacity of these films significantly resulted in an improved wound healing mechanism. Inpanya et al., formulated aloe gel-loaded films that are based on fibroin for diabetic wound management [80]. The mechanical characterizations of wet films exhibited a tensile strength of 18.3 ± 1.3 MPa and elongation at break of 1.9 ± 0.1% that can be beneficial for handling during wound dressing application. The cell proliferation studies showed high cell proliferation and adhesion of skin fibroblasts on films, indicating non-toxicity. The in vivo studies using STZ-induced diabetic rats demonstrated that the wounds treated with the aloe gel-loaded films were significantly smaller by day 7 after surgery than untreated diabetic wounds. The histology assessments of repaired diabetic lesions exhibited the fibroblast distribution and collagen fibre organization similar to lesions in normal rats [80]. Aloe gel in the films induced the fibroblast distribution and the collagen fibre organization for accelerated wound recovery. 

Wu et al., reported silk fibroin-chitosan films encapsulated with adipose-derived stem cells (ADSCs) for diabetic wound care. The wound healing studies in vivo employing diabetic SD rats showed that the tissue of the wound, which was wrapped in the ADSCs-loaded silk fibroin-chitosan films, almost redevelop close to the normal tissue [81]. The encapsulation of the stem cells into the films resulted in enhanced wound healing, which may be due to their similarities with the skin cells. Tong et al., formulated PVA-cellulose nanocrystal films incorporated with curcumin for antimicrobial diabetic wound care. The transmission electron microscope (TEM) analysis of the films demonstrated needle-like cellulose nanocrystals with a mean length of about 159 nm. The mechanical characterization study of curcumin-loaded films demonstrated a tensile strength of 17.13 ± 1.8 MPa and Young’s modulus of 883 ± 140 MPa. The antimicrobial analysis of curcumin-loaded films showed superior broad-spectrum antimicrobial efficacy against *E. coli*, MRSA, *Proteus mirabilis*, *Yersinia sp.,* and *P. aeruginosa*. The in vivo studies using STZ-induced diabetic SD rats showed that there was a significant wound reduction from day 7 post-surgery on wounds dressed with curcumin-loaded films when compared to plain films wound dressings [82]. The excellent mechanical properties and antibacterial activity of the films contributed to the fast diabetic wound healing process because of the presence of PVA and curcumin, respectively. The in vivo studies of retinoic acid-encapsulated solid lipid nanoparticles enclosed by chitosan films reported by Arantes and co-workers using STZ-induced diabetic mice showed an increase in wound contraction after the 5th-day post-surgery when compared to the blank films, indicating that retinoic acid-encapsulated solid lipid nanoparticles enclosed by chitosan films accelerate skin wound closure in diabetes [83]. The features of lipid nanoparticles such as the nanosized structure are the main reason for the rapid wound closure. 

Arul et al., developed collagen-based films encapsulated with biotinylated GHK peptide for diabetic wound dressing. The morphological assessments using SEM of cell culture showed that fibroblasts grown on films were elongated, spindle-shaped, and bipolar in nature, indicating good cell growth and migration. The in vivo studies employing diabetic rats showed that the wounds were almost closed by 99.39% when treated with biotinylated GHK-loaded films on day 21 when compared to 69.49% wound closure for plain films [84]. The use of collagen, one of the ECM constituents to prepare the films promoted the rapid wound healing process. Kim et al., designed PVP-PVA film-forming hydrogels when applied at wound site loaded with sodium fusidate. The film-forming time of hydrogels was between 5 and 6 min. The mechanical performance characterizations of film-forming hydrogels exhibited high Young’s modulus and tensile strength that can be beneficial for wound management. The in vivo experiments on STZ-induced diabetic rats showed that the film-forming hydrogels possess a higher wound reduction rate when compared to control and the commercial product [85]. The good mechanical performance and loading of sodium fusidate in the films provided a suitable environment for diabetic wound healing. The selenium-loaded cellulose films reported by Song and co-workers demonstrated the accelerated healing process of cutaneous wounds in diabetic STZ-induced SD rats via stimulation of angiogenesis and the glucose receptor signalling pathway [86].

Membranes are wound dressings with a similar structure as films. There are several functions of membranes that make them superior as compared to films. The benefits of using polymer-based membranes in wound management include their ability to absorb excess exudate, maintain an appropriate moist environment for the wound healing process, retain biological fluids under pressure, need infrequent dressing changes, reduce the disruption of the wound bed, present potential cleaning activity [87]. Furthermore, membranes demonstrate good mechanical properties such as flexibility, comfortability, softness, and stretchability [87]. Some researchers reported the potential of polymer-based membranes loaded with bioactive agents for diabetic wound care management. Most of the polymer-based membranes have been electrospun to further enhance their physicochemical and biological properties. Augustine et al., prepared electrospun poly(3-hydroxybutyrate-co-3-hydroxyvalerate) (PHBV)-based membranes loaded with cerium oxide nanoparticles for the treatment of diabetic wounds [88]. The FTIR and XRD spectrum confirmed the successful loading of nanoparticles into the membranes. The mechanical characterization of membranes exhibited tensile strength, elongation at break, and modulus of 4.38 ± 00.36 MPa, 65 ± 8%, and 11.18 ± 3.14 MPa, respectively, that are in the range of human skin mechanical performance. The in vivo studies using diabetic rats displayed that a higher wound healing mechanism was observed in nanoparticles-loaded PVBV membranes when compared to plain membranes, which might be due to the antioxidant property of cerium oxide nanoparticles. In addition, the histopathological assessment exhibited a significant enhancement in cell infiltration and granulation tissue development in nanoparticle-loaded membranes dressed in diabetic wounds than those dressed with bare membranes on the 30th day of healing [88]. The excellent mechanical properties, improved cell infiltration, and granulation tissue development significantly resulted in enhanced wound healing in vivo. 

The connective tissue growth factor-encapsulated electrospun PVA-PLA hybrid membranes formulated by Augustine demonstrated high cell proliferation and migration of fibroblasts, keratinocytes, and epithelial cells with potential angiogenesis, indicating that these materials can be employed as outstanding wound dressing membranes for managing diabetic lesions and other chronic ulcers [89]. The encapsulation of growth factors promoted vital cellular processes that promote improved healing of diabetic wounds. Lobmann et al., reported HA membranes encapsulated with human keratinocytes for clinical management of diabetic foot wounds. The outcomes demonstrated that 11 out of 14 type-2 diabetic patients with non-healing wounds treated with these membranes were completely healed after 64 days [90]. Keratinocytes, an important skin cell significantly stimulated fast wound healing of the diabetic injuries. Lee et al., fabricated electrospun nanofibrous PLGA-collagen scaffold membranes loaded with glucophage for diabetic wound management. The mechanical analysis displayed that the loading of glucophage into the membranes insignificantly reduced mechanical performances (tensile stress and elongation at break) of pristine membranes. The water uptake studies exhibited that the water content of the membranes increased with time. The in vivo wound healing studies using diabetic STZ-induced SD rats demonstrated that the Glucophage-loaded membranes significantly accelerated wound healing process with high cell migration and proliferation of keratinocytes on full thickness wounds when compared with plain membranes and gauze sponges [91]. The electrospinning of the membranes and the presence of collagens resulted in a scaffold that mimics ECM and providing a suitable environment for wound healing processes. Lee et al., also reported the biodegradable PLGA-based membranes loaded with metformin. The in vitro drug release experiments at physiological studies exhibited that high concentrations of metformin were released from the nanofibrous membranes for more than 21 days. Furthermore, the wettability studies displayed that nanofibrous metformin-encapsulated membranes were more hydrophilic and had a greater water uptake capacity than plain membranes. The in vivo studies demonstrated that the membranes significantly enhanced wound healing and re-epithelialization in diabetic rats in comparison to the control [92]. The prolonged drug release mechanism of metformin from membranes resulted in improved diabetic wound healing and re-epithelization. 

Ren et al., fabricated PLLA-based electrospun fibrous membranes encapsulated with dimethyloxalylglycine-loaded mesoporous silica nanoparticles for diabetic wound treatment [93]. The wettability analysis of membranes demonstrated a water contact angle of 70° when loaded with nanoparticles, indicating that mesoporous nanoparticles significantly improve the hydrophilic nature of membranes. The drug release studies displayed that dimethyloxalylglycine-loaded mesoporous silica nanoparticles were initially rapidly released from membranes for 48 h, followed by slowly sustained drug release in vitro. The in vitro experiments demonstrated that nanoparticle-loaded membranes possess the ability to promote the proliferation, migration, and angiogenesis-related gene expression of human umbilical vein endothelial cells compared to the pristine membranes. The in vivo studies using STZ-induces diabetic wounds demonstrated that the wounds dressed with plain membranes and dimethyloxalylglycine-loaded mesoporous silica nanoparticles-encapsulated membranes possessed wound closure ratios of 94% and 97%, respectively, considerably higher than that of the untreated wounds (84%), after 15 days [93]. The dimethyloxalylglycine-loaded mesoporous silica nanoparticles significantly induced a fast wound healing process of STZ-induces diabetic wounds.

Liu et al., prepared cellulose acetate-zein composite nanofiber membranes incorporated with sesamol for diabetic wound treatment. The histological studies demonstrated that the membranes loaded with the high content of sesamol resulted in significantly promoted development of myofibroblasts by enhancing transforming GF-β signaling pathway transduction, and stimulated keratinocyte growth by hindering chronic inflammation in wounds, thus improving the wound healing process in diabetic mice [94]. Zheng and co-workers formulated PLGA-cellulose nanocrystals nanofiber membranes loaded with neurotensin for diabetic wound care. The in vitro drug release profile demonstrated sustained release of neurotensin from nanofibrous nanofiber membranes. The wound healing experiments showed that full-thickness wounds in diabetic rats were faster closed when treated with neurotensin-loaded hybrid nanofiber membranes in comparison with plain nanofiber membranes [95]. The incorporation and sustained drug delivery of neurotensin from nanocrystals nanofiber membranes stimulated an increased rate of wound healing. 

The mechanical characterizations of polymer-based films or membranes loaded with various bioactive agents displayed good mechanical properties that can be useful for handling during wound dressing application in diabetic injuries. Most of the mechanical properties are similar to those of human skin, showing that these materials can be compatible with the skin to support wound healing mechanisms. The histological studies of drug-loaded films/membranes displayed superior formation of granulation tissue as well as interesting reepithelization in diabetic wounds, which can lead to fast wound recovery of diabetic wounds. The in vitro drug release studies exhibited sustained and controlled mechanisms of drugs from films. Furthermore, the in vivo wound healing experiments exhibited accelerated wound closure for drug-loaded films/membranes than pristine materials, free drugs, and controls. The limitation of films and membranes is their inability to absorb a large amount of wound exudates, making them inappropriate for high exuding diabetic wounds. 

### 5.3. Hydrogels

**Figure 5:**
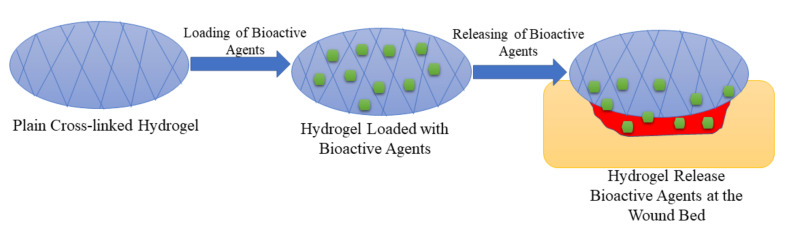
Cross-linked hydrogel loaded with bioactive agents.

Hydrogels have attracted much attention in various biomedical applications in the past decades. They are 3-dimensional networks of cross-linked polymers (Figure 5) which consist of more than 90% moisture content and are fabricated naturally, or through synthesis, via chemical or physical crosslinking methods [96]. They have similarities with living tissues, adhesive nature, and they are malleable, and these characteristics make them considered as the best choice for wound dressing. Hydrogel dressings can accelerate the wound healing process since they can cool the wound through a gaseous exchange, reduce the pain by absorbing wound exudate, and preventing infections, and they can maintain a moist environment for cell migration. Furthermore, hydrogels can act as a delivery system that minimizes side effects and drug toxicity [97,98,99]. There are several reports on the formulation of polymer-based bioactive hydrogels to improve the therapeutic effects of the currently used wound dressing materials to accelerate the wound healing process. 

Wang et al., fabricated promising self-healing polypeptide-based hydrogel (denoted as FHE@exo hydrogel) with pH-responsive long-term exosomes release using Poly-ε-L-lysine (EPL), oxidative HA (OHA), and Pluronic (denoted as FHE hydrogel) by loading adipose mesenchymal stem cells (AMSCs)-derived exosomes through electrostatic interaction between EPL and exosomes [100]. The in vivo studies of FHE@exo hydrogel, FHE hydrogel, and free exosomes were used with saline as a blank control demonstrated that all of them showed decreased diabetic wound size in all treated wounds within 14–21 days after surgery and FHE@exo hydrogel showed faster contraction rates with 88.67 ± 6.9% closure rate on day 14, compared to 36.3 ± 10.4% (saline), 64.3 ± 9.8% (FHE hydrogel) and 76.3 ± 3.2% (exosomes), respectively and at day 21 diabetic injuries treated with FHE@exo hydrogel were completely closed with remarkable hair growth [100]. The loading of exosome into hydrogels significantly improve the wound healing process in vivo. 

Xu et al., formulated hybrid injectable hydrogel for diabetic wound healing management using thiolated HA (HA-SH) and hyperbranched multi-acrylated PEG macromers (HP-PEGs) as retention platform and stem cell delivery via a thiolene click reaction under physiological conditions [101]. This hydrogel displayed stable mechanical properties, antifouling properties, and the encapsulation of adipose-derived stem cells (ADSCs) resulted in improved regenerative capabilities leading to the enhanced wound healing process. Additionally, injuries dressed with HP-PEG/HA-SH/ADSC hydrogel displayed enhanced regenerative features like they have much thicker dermis (95.2% ± 1.7) compared to the only cell-treated wounds (75.5% ± 7.3) and no treatment wounds (42.2% ± 3.4). Also, their wound closure rate (1.9 fold) was better than the only cell-treated wounds (1.3 fold) and no treatment wounds (1.4 fold) at days 11 and 21 post-surgery [101]. ADSCs induces good wound healing effects of the hydrogels by promoting promising skin regeneration capability. 

Zhao et al., developed pH and glucose dual-responsive injectable hydrogels for diabetic foot ulcer (DFU) via Schiff base cross-linking methods (pH-responsive benzoic acid imine) and glucose-responsive phenylboronate ester for drug delivery using PVA, benzaldehyde-capped PEG (OHC-PEG- CHO), and phenylboronic-modified chitosan (CSPBA) as starting materials [102]. These hydrogels were encapsulated with fibroblasts and insulin as selected cells and drug simultaneously because they are good at improving skin repair and wound healing by lowering glucose levels in the diabetic area, accelerating the growth of hair follicles, microvessels, the formation of epidermis, etc. and these hydrogels were evaluated in vivo using SD induced diabetic wound rat model [102]. The rats were treated with PBS as control, neat hydrogel, insulin hydrogel, and insulin/L929 (fibroblast cells) hydrogel, insulin/L929 hydrogels showed enhanced wound closure rate (70 ± 11%) compared to the three groups on day 6 (i.e., PBS (46 ± 11%), neat hydrogel (60 ± 7%), insulin hydrogel (63 ± 4%)) with significant wound closure rate (92 ± 8%) on day 12. The co-loading of insulin and fibroblast cells into the hydrogels is a promising approach to develop wound dressing materials because of their capability to accelerate wound repair and improve the wound healing process [102].

Da Silva et al., fabricated a HA-based spongy hydrogel encapsulated with human adipose stem cells (hASCs) to enhance the therapeutic effect for DFU wound healing and evaluated them against diabetic mice full-thickness wound [103]. These hASCs-loaded gellan gum-HA spongy hydrogels were precultured in selected and standard neurogenic conditioning media. These hydrogels were considered a promising material to manage DFU because of their capacity to generate constructs to control angiogenesis and inflammation and stimulate neo-innervation. The wound closure rate of these hydrogels was studied, and the diabetic mice wounds were treated with GG-HA spongy hydrogel, hASCs-GG-HA condition to neurogenic medium (cond_A_hASCs-GG-HA) spongy hydrogel, hASCs-GG-HA spongy hydrogel, and a control. After two weeks of transplantation, wounds were still open, but (cond_A_hASCs-GG-HA) spongy hydrogels (83.7 ± 11.2%) displayed improved wound closure rate compared to other groups i.e., GG-HA spongy hydrogels (83.7 ± 11.2%),hASCs-GG-HA spongy hydrogels (83.7 ± 11.2%) and control (83.7 ± 11.2%), respectively. Furthermore, the majority of wounds treated with spongy hydrogels containing stem cells were closed after four weeks of transplantations. Therefore, the incorporation of stem cells into hydrogels is a promising strategy to improve the therapeutic effect on diabetic wound healing [103].

Yoon et al., reported horseradish peroxidase (HRP)-catalyzed sprayable gelatin hydrogels (GH) as a drug delivery system of chemotactic cytokines (cell-recruiters) for diabetic wound healing [104]. Two types of chemokines (i.e., macrophage inflammatory protein-3a (MIP-3a) and interleukin-8 (IL-8)) were encapsulated into GH during their in situ crosslinking. The therapeutic effects using streptozotocin (STZ) - induced diabetic mice were reported. The wounds dressed with the chemokine-loaded GH displayed enhanced wound healing activity compared to injuries treated with GH alone or no treatment with improved collagen deposition and neovascularization/re-epithelialization in vivo. The STZ-induced diabetic mice wound sites were treated with MIP-3a-loaded GH, IL-8-loaded GH, controls, and GH only. On the 7th day, it was wounds treated with IL-8-loaded GH (60 ± 9%) which displayed significant improvement in wound closure, and MIP-3a-loaded GH (37 ± 7%) treated wounds showed a similar level of wound closure as injuries dressed with control (34 ± 8%) and GH only (43 ± 3%). Additionally, at day 14, MIP-3a-loaded GH treated wounds displayed improved wound closure than those treated with GH only or controls, but injuries treated with IL-8-loaded GH were remarkable because, on day 10, and enhanced wound closure (83 ± 3%) was visible and showed complete closure on day 14 [104]. The incorporation of horseradish into the hydrogels significantly promoted improved diabetic wound healing process by stimulating collagen deposition and neovascularization/re-epithelialization.

Three are reports on the therapeutic activities of *Blechnum orientale Linn*. (*B. orientale*) (obtained from fern extracts), including the treatment of ulcers, sores, topical wounds, blisters, boils, anti-glucosidase, human colon cancer cells, fever, and antibacterial activity against gram-positive bacteria. Lai et al., studied the treatment of diabetic ulcer wounds using fern extracts (*B. orientale*) [105]. The fern extracts were loaded into sodium carboxymethylcellulose hydrogels (NaCMC). Their wound healing effects on ulcer wounds of STZ-induced diabetic mice and the wound size was measured for 14 days. The STZ-induced diabetic mice wound sites were treated with five groups and the *B. orientale*-loaded hydrogels exhibited improved therapeutic effects with high concentrations (4%) of *B. orientale* extracts loaded to the optimized hydrogel formulation. The hydrogel accelerated the wound healing with complete re-epithelization by an average of 2 days and wound closure on day 12 compared to low concentrations (2%) extracts and controls, which displayed a wound closure at day 14. However, since these hydrogels are used as a delivery system for the extracts, sometimes it was observed that wounds treated with high concentration (4%) extract loaded hydrogels extended the inflammation resulting in a temporal increase in wound size, but it was not observed in those treated with 2% concentration extract loaded hydrogels. These fern extracts are potential therapeutics for treating diabetic ulcers [105].

Kaisang et al., reported injectable Pluronic F-127 hydrogels encapsulated with adipose-derived stem cells (ADSCs) as drug delivery systems to enhance diabetic wound healing. They evaluated them in vivo utilizing an STZ-induced diabetic model in rats [106]. These hydrogels seeded with the cells displayed good biocompatibility, thermosensitivity which contributed to improved angiogenesis and enhanced cell proliferation at the wound site and also improved wound closure rate resulting in the acceleration of granulation tissue repair. Furthermore, wound closure rate was studied, and wounds of rats were treated with ADSCs-Pluronic-F127 hydrogels, PBS control, ADSCs alone, and Pluronic F-127 alone for a period of 14 days (0-3-7-10-14), at day 3, there was no significant change in the wounds, but on days 7 and 10, the wounds treated with ADSCs-Pluronic-F127 hydrogels displayed improved closure rate as the size of the wound decreases compared to those treated with other three aforementioned groups with *p* < 0.05 and at day 14 wounds treated with ADSCs-Pluronic-F127 hydrogels were almost completely closed with others treated with PBS control (20.5%), ADSCs alone (10%) and Pluronic alone (18.8%) were percentage less complete. It was further reported that vascular endothelial growth factor, levels of the messenger RNA expression of key angiogenesis growth factor, transforming growth factor-beta 1, and key wound healing GF were enhanced on ADSCs-Pluronic-F127 hydrogels treated wounds compared to untreated wounds [106]. The encapsulation of ADSCs into polymeric hydrogels significantly enhanced diabetic wound healing. 

Moon et al., reported hydrogel complex containing allogeneic adipose-derived stem cells (AASCs) as a potential treatment of DFU in clinical studies. These AASC-loaded hydrogels (30 patients) were applied as sheets to the DFU wound sites together with polyurethane film (29 patients) as a control on a total of 59 patients to evaluate their therapeutic effect on DFU for 12 weeks, and neither of them (hydrogel or control) were applied weekly [107]. It was further reported that at week 8, the complete wound closure was observed on 73% of patients treated with AASC-hydrogels compared to 43% of those treated with polyurethane films. A significant improvement was observed on week 12 with 82% of the patients treated with AASC-hydrogels showed complete wound closure compared to 53% of those treated with control [107]. The loading of AASCs improved the wound healing of the hydrogels in clinical trials. 

Li et al., fabricated hydroxyapatite/chitosan composite hydrogels encapsulated with exosomes as a material to treat diabetic wounds (HAP–CS–SMSCs-126–Exos hydrogels) [108]. These hydrogels were tested in vivo, and they displayed improved features, including expedite collagen maturity, accelerate angiogenesis, and wound surface re-epithelialization, and the presence of chitosan; hydroxyapatite and exosomes were responsible for the enhanced therapeutic effect. The wounds treated with pristine hydrogels, exosome-loaded hydrogels, and controls, did not show any remarkable difference during the post-operation period but the wound size of the wounds dressed with hydrogels was smaller than those of the control group. Furthermore, a notable difference was observed after 14 days post-operation in which wounds treated with exosome-loaded hydrogels fully recovered compared to those treated with pristine hydrogels, which were almost completely closed and the untreated wound was still injured (open). Although the difference was not observable, the loading of exosomes into hydrogels promoted a faster-wound closure rate than the other groups suggesting that these hydrogels are useful for treating diabetic chronic wounds [108].

Zhu et al., fabricated antioxidant thermo-responsive hydrogel for DFU care management. These hydrogels enhanced the dermal wound healing process in diabetes by releasing stromal cell-derived factor-1(SDF-1) [109]. They prepared these hydrogels using SDF-1 together with PEG-citrate-co-N-isopropylacrylamide) (PPCN) and evaluated their therapeutic effects in a diabetic murine splinted excisional dermal wound model. It was observed that PPCN affects the release of SDF-1, with an increase in PPCN concentration decreases the SDF-1 release rate. Furthermore, wounds treated with this hydrogel showed improved healing activity compared to injuries treated with other groups (SDF-1 only, PPCN only, and PBS) as they took a shorter time (24 days) to completely closed the wound than others with the highest density of perfused blood vessels, improved epithelial maturation, and granulation tissue production [109]. Loading of SDF-1 into the hydrogels im resulted in an improved wound healing process of the wounds. Veerasubramanian et al., prepared hydrogels for diabetic wounds using an ethanolic extract of Avena sativa (OAT), human hair proteins (KER), and konjac glucomannan (KGM) as a starting material and in vivo evaluated them in a diabetic rat excision wound model. These non-toxic, cost-effective hydrogels exhibited enhanced therapeutic effects due to the good properties of the material used. For instance, KER is biodegradable, biocompatible, etc., and can also support collagen expression, keratinocyte migration, fibroblast attachment, and proliferation. On the other hand, OAT can prevent prolonged inflammation in chronic wounds since it contains antioxidant moieties. Therefore, KGM + KER + OAT hydrogels showed no remarkable cytotoxicity against NIH/3T3 fibroblasts and enhanced wound healing activity because of their natural-based components when compared to KGM +KER hydrogels [110].

Thangavel et al., fabricated chitosan-based hydrogels encapsulated with L-glutamic acid (LG) to enhance the diabetic wound healing process. These hydrogels exhibited good thermal stability, controlled biodegradation, good swelling, and smooth surface morphology, even the addition of LG did not change the biocompatibility of these hydrogels instead, they accelerate the wound healing process with diabetic wounds treated with these hydrogels took 16 days to recover compared to wounds treated with plain hydrogel (20 days), and control (26 days) when evaluated on diabetic rats, in vivo. The crosslinking methods and the addition of LG promoted collagen deposition and accelerated vascularization, resulting in enhanced therapeutic effects of these LG + CS hydrogels for diabetic wounds [111]. Curcumin exhibit several therapeutic activities such as anticancer, antioxidant, wound healing, antimalarial, etc., but its molecules are bioavailable unstable in vivo; hence Liu et al., developed thermosensitive hydrogels in the form of gelatin microspheres (GMs) containing a nanodrug of curcumin for improved diabetic wound healing. It was evaluated on streptozotocin-induced diabetic mice [112]. The bioavailability of curcumin was improved by preparing curcumin nanoparticles, and these nanoparticles were enclosed to the GMs and loaded into the hydrogel. The successful development of these CPNs promoted cell migration which rapid skin wound healing [112]. 

Masood et al., prepared chitosan-PEG hybrid hydrogels incorporated with Ag nanoparticles to improve diabetic wound healing, and they further evaluated them in wounds on diabetic-induced rabbits [113]. The in vitro drug release profile displayed a controlled release of the Ag nanoparticles from the hybrid hydrogels, indicating that the slow controlled release of the nanoparticles can accelerate the wound healing process. However, for seven days, these hydrogels displayed sustained and slow release of nanoparticles resulting in slow biodegradation of these hydrogels. Additionally, diabetic wounds treated with these hydrogels showed improved wound healing capability with improved antioxidant and antimicrobial properties by exhibiting a higher degree of swelling, higher porosity, and higher WVTR compared to wounds treated with chitosan-PEG hydrogel only, suggesting that these Ag nanoparticle-loaded hydrogels can promote diabetic wound healing [113]. The controlled release of Ag nanoparticles from the hydrogels significantly promoted improved healing of the diabetic wounds in vivo.

Xiao et al., developed copper metal-organic framework-hydrogel to promote the diabetic wound healing process. Copper ion promote wound repair by inducing angiogenesis, but its application needs to be repeated several times to enhance the healing of a diabetic wound, and this can lead to a high level of toxicity to the wound site [114]. Hence, hydrogels were developed to reduce the toxic levels of copper ions by controlling the release of copper ions or oxides and accelerate wound healing. The antioxidant thermoresponsive citrate-based hydrogel loaded with copper metal-organic framework nanoparticles (HKUST-1 nanoparticles) displayed a slow copper ion release rate and prevented toxicity. However, HKUST-1 NPs decompose in protein solutions, thus they were prepared by implanting them in poly-(polyethyleneglycol citrate-co-N-isopropylacrylamide) hydrogel (H-HKUST-1) which were characterized and evaluated in a splinted excisional dermal wound diabetic mouse model, in vivo. The H-HKUST-1 hydrogel protected the nanoparticles from decomposing, and released the copper ions slowly, resulting in reduced apoptosis and cytotoxicity with enhanced dermal cell migration, and improved wound closure rates [114].

Masters et al., investigated the therapeutic effects of nitric oxide on diabetic wound healing by developing nitric oxide (NO)-loaded PVA hydrogels [115]. The in vitro experiments exhibited that over 48 h NO was released from NO hydrogel and there was no change in fibroblast growth associated with this hydrogel but the ECM was produced more in cells treated with NO hydrogels compared to untreated cells. In vivo studies on diabetic mice were conducted using different doses of NO (low = 0.5 mM and High = 5 mM) and the wound closure rate of wounds treated with controls and those treated with NO hydrogels was similar. However, NO hydrogel wounds were still wider than control wounds on day 8, but this trend was not observed on days 10 and 13 of the treatment. Moreover, the histology analysis showed that after wound closure at days 8 and 15, scar tissue thickness and granulation tissue thickness within the wounds of the diabetic mouse were enhanced on wounds treated with 5 mM NO hydrogels. The findings suggest that the presence of NO in the polymeric hydrogels can be considered as a potential strategy to accelerate wound healing process [115]. Tokatlian et al., developed porous HA hydrogels for localized non-viral DNA delivery to enhance therapeutic effects. They used porous (100 μm) and non-porous (60 μm) HA-MMP hydrogels loaded with pro-angiogenic (pVEGF) plasmids or reporter (pGFPluc) to investigate gene delivery using the diabetic mouse. Non-porous hydrogels showed a mechanical barrier to wound closure because they did not degrade. On the other hand, porous hydrogel promoted a faster wound closure rate than non-porous hydrogel. The presence of pDNA/PEI polyplexes enhanced the formation of granulation tissue even when the DNA did not encode for an angiogenic protein [116]. The porosity of HA hydrogels played a significant role in promoting wound healing by inducing angiogenesis.

Zhang et al., formulated poly (γ-glutamic acid)/heparin/chitosan composite hydrogels loaded with superoxide dismutase for the treatment of diabetic wounds. The in vitro cytocompatibility studies exhibited good cell migration and proliferation of 3T3 fibroblasts when cultured with composite hydrogels showing cell viability of higher than 70%, confirming non-toxicity of hydrogels. The in vivo experiments using the diabetic rat model exhibited an accelerated wound healing process when the wounds were treated with superoxide dismutase-loaded hydrogels when compared to control and plain hydrogels. These wound healing results are attributed to the wound-healing effects of chitosan by promoting cell proliferation, and loading of superoxide dismutase by decreasing ROS production at the wound bed [117]. The gelatin methacryloyl injectable hydrogels designed by Chen et al., were loaded with cerium-containing bioactive glass nanoparticles. These hydrogels significantly reduced colony numbers of both *S. aureus* and *E. coli*, revealing their potential for the treatment of bacterial-infected diabetic wounds. The in vivo wound healing studies using the full-thickness skin defect model of diabetic showed that the wound closure time in the groups of injectable hydrogels loaded with nanoparticles was faster than the blank hydrogels and the control group [118]. Shi et al., prepared chitosan-dextran hydrogels loaded with Ag nanoparticles for diabetic wound treatment. The antimicrobial experiments of Ag nanoparticle-loaded hydrogels demonstrated broad-spectrum and long-lasting antibacterial activity. These hydrogels exhibited rapid wound closure, indicating their superior healing efficacy to promote granulation tissue development, fibroblast migration, and angiogenesis [119].

The polymer-based hydrogels exhibit high porosity that can provide high swelling capacity, cell growth, and cell migration to stimulate the wound healing process of diabetic wounds. The hydrogels loaded with bioactive agents demonstrated the ability to promote significant processes that include reepithelialization and the development of granulation tissue, which are important for the recovery of diabetic wounds. The in vitro drug release experiments exhibited a controlled release of the loaded drugs from the hybrid hydrogels. The loading of bioactive agents onto the polymeric hydrogels significantly improves their therapeutic outcomes that lead to a fast wound healing process of diabetic injuries in the in vivo series, indicating that these hydrogels are auspicious candidates that can be used for the treatment of diabetic injuries. However, the polymeric composition of hydrogels must be considered because hydrogel dressings that are only formulated from natural polymer can lead to poor mechanical properties, making these dressings to be non-biocompatible with the human skin. The hydrogel wound dressings are not appropriate for low exuding wounds due to their high porosity and water uptake that can result in wound dehydration. 

### 5.4. Foams and Wafers

**Figure 6:**
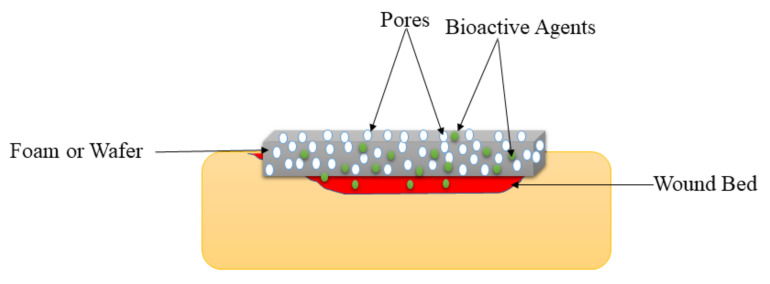
Foam or wafer on the wound bed.

Foams are solid porous wound dressings (Figure 6) that are made of hydrophobic and hydrophilic foam with bioadhesive boundaries [42]. The external hydrophobic layer protects the injury from the liquid but allows gaseous exchange and water vapor permeation. These wound dressings can be sterilized and applied on injuries without resulting in pains to the patient if their parameters (such as mechanical properties, density, and thickness) are appropriately tailored. Foam wound dressings possess several advantages such as improved gaseous exchange, protect the wound from maceration, offer suitable moisture for the fast wound healing process, and absorb large amounts of exudate, making them appropriate for the management of burns, diabetic ulcers, traumatic wounds, etc. [120]. The shortcoming of foam wound dressing materials is that they are inappropriate for dry wounds or injury with low exudates and dry scars [121]. Pyun et al., formulated PU-based foams incorporated with recombinant human epidermal growth factor (rhEGF) for diabetic wound treatment. The FTIR spectrums confirmed the successful fabrication of the PU foam dressings. The water vapor transmission experiments of foams demonstrated a WVTR of about 3000 g/m^2^/day, which is close to ideal wound dressings (2000–2500 g/m^2^/day) [122].

The cytotoxicity analysis in vitro exhibited very high cell proliferation and viability of CCD986-skin human fibroblast cell lines and HaCaT human keratinocyte when incubated with rhEGF-loaded foams, suggesting excellent biocompatibility of PU foams. The in vitro release profile displayed rapid release of rhEGF from the surface of foams in the first 24 h, followed by plateau release for 7 days. The in vivo studies using STZ induced diabetic SD rats showed that the full-thickness wounds were almost completely closed by more than 97% when treated with rhEGF-loaded foams. The histological analysis demonstrated that the diabetic wounds were completely resolved by regenerating the epithelial cell in the rats on day 21 after wounding [122]. The moderate WVTR and release profile promoted enhanced healing of the diabetic wounds by inducing epithelial cell regeneration. Coutts et al., conducted clinical studies of PVA foam wound dressings co-loaded with gentian violet and methylene blue for bacterial-infected diabetic wounds [123]. The outcomes of these studies presented enhancements in surface critical colonization and pain score at the end of the assessment period in some patients, especially in patients with DFUs. Furthermore, decreasing wound size was observed in 8 of the 14 patients at week 4 [123]. The other clinical studies reported by Moon et al., demonstrated that the wounds in diabetic patients dressed with Ag-incorporated PU foams were restored in 15.6 ± 3.8 days while those treated with plain foams healed in 14.4 ± 2.2 days, revealing that the presence of silver in the foams delayed the epithelialization of the diabetic injuries in patients. However, the difference was statistically significant in this study [124]. 

Choi et al., fabricated PU foams loaded with Ag nanoparticles and rhEGF for bacteria-infected diabetic wound management [125]. These foam wound dressings significantly demonstrated fluid retention, excellent absorbency, and fluid handling features. SEM micrographs exhibited that the PU foams demonstrated a relatively uniform pore size that ranges between 200–400 µm and it was not affected by the incorporation of bioactive agents, suggesting that these foams can provide high cell granulation rate and proliferation with an excellent gaseous exchange during wound healing. The in vitro cytotoxicity analysis utilizing MTT assay exhibited the high cell viability of L929 mouse fibroblasts when cultured with dual bioactive agent-loaded foams. The antimicrobial analysis using the inhibition zone method displayed that the PU foams loaded with Ag nanoparticles and rhEGF exhibited outstanding antibacterial efficacy (high inhibition zone) against *E. coli* and *S. aureus,* while unloaded foams did not display any inhibition effects. The in vivo experiments utilizing diabetic Balb/b mice demonstrated that injuries wrapped with the foams loaded with both Ag nanoparticles and rhEGF demonstrated excellent healing after 5 days of treatment than the gauze, suggesting a synergistic effect of incorporating bioactive agents together with growth factors [125].

Gunal et al., reported the comparison of silver-coated foams (GranuFoam Silver) and uncoated foams (GranuFoams) for the management of DFU in clinical studies. The results demonstrated that the diabetic wounds in patients treated with silver-coated foams showed an average surface area of 41.55–36.03 cm^2^ before treatment and 7.64–3.91 cm^2^, 10 days after treatment when compared to plain foam that demonstrated 18.40–23.48 cm^2^ wound surface area after 25 days [126]. Bai et al., formulated silk fibroin foams wound dressings enriched with gastrodia elata and tea tree oil for diabetic wound management [127]. The SEM images of the foams showed highly porous morphology with porosity that ranges between 40% and 80%. The in vitro biocompatibility studies of bioactive agent-loaded foams exhibited 90–100% cell viability of 3T3 fibroblast cells, indicating that these foams are non-toxic to the skin cells. In vitro antioxidant studies of foams significantly displayed more than 70% reduction in nitrite production, indicating excellent anti-inflammatory efficacy. In vivo experiments demonstrated that all plant extract–loaded silk fibroin foam wound dressings significantly accelerated wound healing and completed full wound closure within 21 days. Furthermore, the histological assessment of regenerative skin tissues demonstrated that the foam wound dressings improve the generation of denser, thicker, and more abundant collagen fibres in the dermis layer [127]. The co-encapsulation of gastrodia elata and tea tree oil resulted in good antioxidant activity with a non-toxic effect on the skin cells and also promoted rapid wound healing. 

Moura et al., reported chitosan-based foams loaded with neurotensin for diabetic wound healing application. The swelling studies demonstrated the fastest swelling rate of chitosan foams, reaching a maximum of 2438% after 5 h. The in vivo diabetic wound healing experiments exhibited that the neurotensin-loaded chitosan foams treatment was significantly more effective in comparison with pristine foam, with a wound reduction of 50% rather than 35% for the non-loaded foams [128]. The combination of chitosan and neurotensin to formulate the foams significantly promoted enhanced wound healing the diabetic injuries. The silver-loaded silicone foams reported by Tong et al., in clinical studies showed that all diabetic ulcers in patients significantly demonstrated positive wound closure and reduction in size in the period that ranged between 3 to 16 weeks. Furthermore, trauma and skin maceration, and clinical signs of infection were absent in the wounds at the end of the period [129]. 

On the other hand, wafers are wound dressings that have similar properties as foams. Wafers are highly porous freeze-dried polymers of determining structure that has been used as solid delivery systems for the wound care management of various chronic wounds. They absorb wound exudates and change into a gel/viscous solution that offers a moist environment for the acceleration of the wound healing process [130]. There are some polymers, such as sodium alginate and xanthan gum, which are employed for the preparation of polymer-based wafers [131]. The features of wafers include their capacity as topical drug delivery systems, their prolonged residence on the wound, mucoadhesive nature, and their ability to be loaded with both soluble and insoluble antimicrobial agents. Their formulation process is essential because poor fabrication processes from poor ratios of materials can lead to non-porous, sticky, and rigid wafers that are not appropriate for wound treatment [130]. 

Ahmed et al., reported lyophilized calcium alginate-based wafers incorporated with ciprofloxacin for microbial-infected diabetic wound management [132]. The SEM results exhibited that the wafer wound dressings were highly porous in morphology with uniform, circular, and large shaped pores encircled by a network of polymeric strands while porosity investigation demonstrated high percentage porosity that ranges between 98.20 ± 0.56 and 88.42 ± 4.03%, which could be beneficial for gaseous exchange during wound healing. The in vitro drug release studies at physiological conditions exhibited initial rapid release of ciprofloxacin followed by sustained drug release that can inhibit and prevent re-infection caused by both bacterial strains. The in vitro cytotoxicity studies showed more than 85% cell viability of human adult keratinocytes when cultured with ciprofloxacin-loaded wafers indicating excellent biocompatibility. The in vitro antimicrobials studies using disk diffusion methods exhibited that the ciprofloxacin-loaded wafers significantly showed excellent antibacterial activity against *E. coli*, *S. aureus*, and *P. aeruginosa*. In contrast, plain wafers did not display any antimicrobial effect. The outcomes from this study demonstrated that loading ciprofloxacin, an antibiotic in the wafers is useful for the management of bacterial-infected diabetic injuries [132]. 

Gadad et al., fabricated and gamma radiation sterilized xanthan gum-based wafers loaded with silymarin for wound care in diabetic patients. The cell migration studies showed that the silymarin-loaded lyophilized wafers successfully retained their capability to overcome the high glucose-induced reduction in endothelial cell migration, indicating that wafers are useful for diabetic wound management [133]. Atia et al., prepared sodium alginate-gelatin wafers loaded with diosmin nanocrystals for diabetic wound healing application. The in vitro drug release profile demonstrated that the diosmin nanocrystals were significantly released in a sustained manner from the wafers for 8 h. The in vivo wound healing experiments of diosmin-loaded wafers utilizing STZ induced diabetic mice showed a fast wound healing process. The histology analysis of the wafer exhibited well-developed granulation tissue, well-organized dermal layers, complete re-epithelialization, and mature collagen bundles [134]. Sustained drug release of diosmin nanocrystals improved the wound healing processes of diabetic wounds. 

The polymer-based foams and wafers loaded with therapeutic agents exhibited high porosity, indicating that these sound dressing materials can offer a high cell granulation rate and proliferation with an excellent gaseous exchange during the wound healing process of diabetic injuries. The WVTR examinations of drug-loaded foams showed moderate WTVR that can provide a suitable moist environment for fast wound closure. The drug release profiles exhibited that the drugs were released in controlled and sustained phenomena from the foams or wafers. The cytotoxicity experiments in vitro displayed high cell viability of skin cells when incubated with drug-loaded foams and wafers, indicating non-toxicity and good biocompatibility. The in vivo studies demonstrated that the diabetic wounds dressed with drug-loaded foams or wafers healed faster than those treated with plain foams or wafers. Although foams and wafers exhibit interesting properties, they are not suitable for less exuding diabetic wounds.

### 5.5. Sponges and Bandages

**Figure 7:**
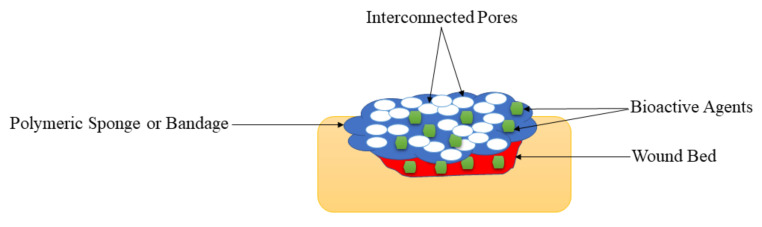
Sponges on the wound bed.

Sponges are wound dressings that possess interconnected porous structures (Figure 7), soft and flexible [135]. Their porous structure influences their high swelling capacity, making them appropriate for the management of exuding wounds. They also support cell migration and high water absorption capability appropriate for providing moisture to the wound bed while protecting the injury from bacterial infections [136]. The sponge wound dressings formulated from PVA, alginate, chitosan, and graphene oxide demonstrated excellent antimicrobial efficacy [137]. Several sponges have been prepared for the delivery of therapeutic agents for the treatment of diabetic wounds. Wang et al., formulated chitosan-cross-linked collagen sponges encapsulated with recombinant human acidic fibroblast growth factors to stimulate the diabetic wound healing process [138]. These hybrid sponges exhibit several advantages required in an ideal wound dressing, such as uniform and porous ultrastructure, in vitro slow release of fibroblast GFs from the sponges, and high resistance to collagenase digestion [138]. The remedial impact of the novel wound dressing comprising fibroblast growth factors on diabetic wound healing was studied in a type 1 diabetic rat model in which hyperglycemia was prompted by a single dosage of STZ and continued for a very long time. The diabetic wound healing was discovered to be significantly enhanced by chitosan-cross-linked collagen sponges loaded with fibroblast growth factor compared to the pristine sponges, revealing the capability of growth factor-loaded chitosan-cross linked collagen sponges wound dressings for diabetic wound healing [138]. The presence of fibroblast growth factors and their sustained drug release mechanism from sponges significantly resulted in accelerated wound healing process.

Anisha et al., formulated HA-chitosan sponges loaded with Ag nanoparticles as a wound dressing for DFUs infected with drug-resistant bacteria. The developed sponges fulfilled the properties of an ideal wound dressing in terms of high porosity, hemostatic potential, swelling, and good biodegradation [139]. The in vitro antimicrobial experiments of the sponges encapsulated with Ag nanoparticles showed excellent antibacterial effects against *S. aureus*, *E. coli*, *K. pneumonia*, *P. aeruginosa,* and MRSA, suggesting their capability to be utilized as a wound dressing for DFUs infected with antibiotic-resistant bacteria. The in vitro cytotoxicity examinations showed that a higher concentration of Ag nanoparticles in the sponges decreased the viability of skin cells when compared to those loaded with low nanoparticle concentration. Therefore, the results reveal that the nanocomposite sponges may be utilized as effective scaffolds for the wound treatment for DFUs infected with antibiotic-resistant bacteria due to their interesting antibacterial activity [139].

Xia et al., successfully formulated chitosan composite sponges incorporated with quaternary ammonium chitosan nanoparticles (TMC nanoparticles) as the wound dressing material to manage chronic injuries. The hydrophobic surface of the sponges exhibits anti-adhesion contaminant activity and is waterproof, while the hydrophilic surface inhibited the growth of bacteria and retained the water-absorbing ability [140]. The incorporation of TMC nanoparticles improved the antibacterial action of the chitosan against gram-positive bacteria *S. aureus* and Gram-negative bacteria *E. coli*. In vivo wound healing evaluation demonstrated that TMC nanoparticle composite sponge stimulates angiogenesis and accelerates re-epithelialization. In vivo anti-infection evaluation reveals the infected injuries dressed with the improved TMC nanoparticle-loaded sponges healed quicker because of the exceptional antibacterial property of TMC nanoparticles. The in vivo wound closure examinations using STZ-induced Kunming mice demonstrated rapid healing of full-thickness wounds dressed with TMC nanoparticle-incorporated chitosan sponges than the plain chitosan wound dressings [140]. The biological activity of the TMC nanoparticles was good antibacterial effects, resulting in improved wound healing of diabetic injuries. 

Mohandas et al., developed chitosan–HA composite sponge loaded with fibrin nanoparticles incorporated with vascular endothelial GFs as a wound dressing for diabetic wounds [141]. The in vitro release experiments showed an initial rapid release of vascular endothelial GFs with factors from the chitosan–hyaluronic acid composite sponge followed by a sustained release until 7 days which is suitable for a wound dressing material. The outcomes show that the developed chitosan–HA-incorporated nanofibrin composite sponge is a good candidate for induced angiogenesis in the wound [141]. These results suggested a potential approach to treat diabetic wound healing because of the sustained release of vascular endothelial GFs. Kondo et al., studied the impact of a sponge-based wound dressing made of HA and collagen and encapsulated with epidermal growth factors (EGFs) in vivo [142]. The epithelialization was facilitated to a greater extent by EGF-loaded sponge wound dressings. The in vivo wound healing studies employing full-thickness wounds in type-II diabetic mice exhibited that the plain hybrid sponges and EGF-loaded hybrid sponges stimulated a decrease in wound size related to blood vascular formation and granulation tissue development, compared with the commercially available artificial dermis [143]. HA and collagen are ECM components that provide a suitable environment for the healing process of diabetic wounds especially when loaded with growth factor, EGFs.

Shi et al., reported chitosan-silk hybrid sponges loaded with GMSC-derived exosomes via freeze-drying technique [144]. Exosomes combined with chitosan sponge enhanced the skin wound healing in an STZ-prompted diabetic rat model. Re-epithelialization and remodelling of ECM and neuronal ingrowth and promoting angiogenesis were observed. These results give a new non-invasive application technique of the exosomes with practical value for skin repair. It also gives new data on the function of the gingival mesenchymal stem cells-derived exosomes in wound healing [144]. Lipsky et al., reported collagen-based sponges loaded with gentamicin for clinical management of DFU. The clinical outcomes demonstrated that the patients treated with gentamicin-loaded sponges showed clinical cures (100%) with pathogen eradication when compared to those who were not treated (70%) after 7 days of wound assessments [143]. The loading of gentamicin in the sponges promoted the wound healing effects of collagen sponges. Momin et al., formulated chitosan-alginate hybrid hydrogel sponges using in situ polymerization procedure. The sponges were incorporated with curcumin and honey for diabetic wound care [145]. The in vitro drug release profile demonstrated that the release of curcumin from sponges was sustained for 20 days, thereby decreasing the frequency of dressing changes required. The formulated sponges were biocompatible and biodegradable. According to the results of the study, sponges loaded with honey-curcumin demonstrated effective and faster diabetic wound healing [145]. The combination of honey and curcumin in sponges resulted in synergistic wound healing effects.

Ti et al., studied the possible utilization of a chitosan-collagen sponge incorporated with thymosin beta 4 to accelerate cutaneous wound healing in STZ-prompted diabetic rats. Chitosan-collagen sponge incorporated with thymosin beta 4 possessed a proper 3D porous structure, swelling property, an optimal biodegrading rate, and better biocompatibility as a wound dressing because it stimulated angiogenesis, promotion of healing, and inhibition of inflammation [146]. The in vivo studies of chitosan-collagen sponge incorporated with thymosin beta 4 exhibited enhanced diabetic cutaneous wound healing with a better dermal reorganization, faster wound re-epithelialization, higher wound vascularization, and also upregulated angiogenic genes and down-regulated inflammatory genes in the wound tissue. Thus, the sponges demonstrated promising potential applications in tissue regeneration, especially in diabetic cutaneous wound treatment [146]. The incorporation of thymosin beta 4 in the hybrid sponges improved the wound healing processes (dermal reorganization, wound re-epithelialization, wound vascularization) that can lead to rapid wound healing. 

On the other hand, polymer-based bandages are wound dressings with similar properties as sponges, and they can be encapsulated with various bioactive agents for diabetic wound care management. Kumar et al., formulated microporous and flexible chitosan-based hydrogel composite bandages loaded with ZnO nanoparticles [147]. The prepared nanocomposite bandages exhibited improved blood clotting, controlled degradation, swelling, and good antibacterial activity. The developed chitosan hydrogel composite bandages displayed approximately 80% porosity of the total bandage volume and were useful in absorbing huge amounts of wound exudate. The in vitro evaluation of cytocompatibility showed that the bandages demonstrated improved infiltration and cell viability. The in vivo wound healing experiments demonstrated an improved healing capacity of chitosan hydrogel/nano ZnO composite bandages [147]. In vivo and in vitro antibacterial evaluation revealed the antibacterial potential of the developed chitosan hydrogel/nano ZnO composite bandages. The in vivo wound closure evaluation in diabetic SD rats demonstrated that these nanocomposite bandages significantly improved wound healing and induced faster collagen deposition and re-epithelialization, making them suitable for DFU [148]. Chitin hydrogel composite bandages loaded with ZnO nanoparticles were reported by Kumar et al., The bandages displayed excellent antibacterial activity and high cell adhesion and migration that are caused by loading of ZnO nanoparticles, demonstrating that these bandages can be useful in diabetic wound treatment [149]. 

Mohanty et al., reported the utilization of EGF-curcumin bandage bioconjugate based on sodium alginate and chitosan. The bandage was loaded with mesenchymal stem cells [148]. The outcomes revealed that the formulated epidermal growth factor-curcumin bandage formed a stable, non-toxic, and biocompatible stage for therapeutic mesenchymal stem cells delivery towards improved wound healing. The outcomes suggest that synergistic incorporation of epidermal growth factor, curcumin, and mesenchymal stem cells in the polymeric bandages can overcome the challenges associated with diabetic wound healing. Therefore biocompatible therapeutic epidermal growth factor-curcumin bandage bioconjugate loaded with mesenchymal stem cells may have good application for diabetic wound healing in the future [148]. Raveendran et al., developed chitosan bandages encapsulated with fluconazole and ciprofloxacin drugs-loaded nanoparticles for a slow sustained release of bioactive agents [150]. The bandages encapsulated with drug-loaded nanoparticles were flexible with sufficient tensile strength and porosity of between 80–85%, which could promote excess exudates absorption in an infected wound. Fluconazole and ciprofloxacin were released from the bandages for 14 days in a sustained manner [150]. Fluconazole and ciprofloxacin drugs loaded bandages exhibited important antimicrobial action on polymicrobial cultures of *E. coli*, *S. aureus,* and *C. albicans* in ex vivo and in vitro. A significant decrease in the microbial load was found upon application of the antimicrobial drug encapsulated chitosan bandages in vivo. The chitosan bandages demonstrated promising potential applications in diabetic wound management [150]. The co-loading of fluconazole and ciprofloxacin resulted in excellent antimicrobial effects that can promote improved wound healing of bacterial-infected diabetic wounds.

The polymer-based sponges and bandages loaded with therapeutic agents displayed high porosity that can promote high cell growth and attachment, which are suitable for the diabetic wound healing process. Also, the initial rapid drug release of bioactive agents from these scaffolds followed by sustained release is appropriate for improving the treatment of diabetic wounds (Table 1 and Table 2). Most of the reported sponges and bandages for the treatment of diabetic injuries were loaded with antibacterial agents (metallic nanoparticles and antibiotics) and they demonstrated excellent antibacterial activity against various antibiotic-resistant bacterial strains, suggesting that these are potential materials for the management of infected diabetic wounds. The encapsulation of bioactive agents into the sponges and bandages significantly accelerated the wound healing process of the diabetic injuries in vivo when compared with diabetic wounds dressed with plain scaffolds. Nevertheless, the very high porosity of polymeric sponges or bandages can result in high uptake of wound exudate and high WVTR that may cause dehydration of diabetic wounds. A dehydrated injury can lead to a delayed wound healing process. 

## 6. Conclusions and Future Perspective 

There are several interesting properties of polymer-based wound dressings that make them useful in diabetic wound management, depending on the dressing type. Some of these properties are high porosity that can promote cell growth and attachment, high water uptake and swelling capacity, moderate WVTR, good mechanical performance, gaseous exchange for cell growth, and providing moisture for fast wound healing. The microbial infections, oxidation stresses, and poor blood flow in diabetic patients result in retarded wound healing process, suggesting that the use of wound dressings without drug delivery properties are not appropriate for the treatment of diabetic wounds. 

Polymer-based dressings loaded with bioactive agents (metallic nanoparticles, antibiotics, stem cells, GFs, vitamins, antioxidants) resulted in accelerated wound healing by promoting vascularization, collagen accumulation, and normal physiological functions. Most dressings loaded with bioactive agents in clinical trials for diabetic wound management were foams. Foams demonstrate good therapeutic outcomes, suggesting that even other polymer-based dressing types that are still in the in vitro and in vivo studies will reach clinical studies soon for the treatment of diabetic wounds. Most studies will result in scaffolds that will reach clinical trials. Those studies include experiments on the analgesic effect of the drug-loaded scaffolds during the diabetic healing process and the long-term toxicity effect of the scaffolds utilized for diabetic wound management. The formulation of drug-loaded wound dressings for the treatment of diabetic injuries using new techniques such as 3D printing; with excellent therapeutic outcomes, outstanding mechanical properties, and good biocompatibility need to be studied. 

## Figures and Tables

**Table 1:** Summary of polymer-based wound dressings loaded with bioactive agents for diabetic wounds.

Types of Wound Dressing	Used Polymers	Loaded Bioactive Agents	Results	Ref
Nanofiber	Gelatin and cellulose	Glybenclamide and metformin	Accelerated wound healing process and good biocompatibility	[57]
Nanofiber	PEG and PCL	EGF	Superior wound healing process	[58]
Nanofiber	Polylactide	Doxycycline	Excellent mechanical performance, antibacterial effects, and excellent diabetic wound healing properties	[59]
Nanofiber	PCL and gum tragacanth	Curcumin	Bead-free morphology and full wound closure on day 15.	[60]
Nanofiber	PU and carboxymethylcellulose	*Malva sylvestris* plant extract	Good diabetic wound healing rate	[61]
Nanofiber	Hydroxypropyl methylcellulose and PEO	beta-glucan	Non-toxic and accelerated wound closure.	[62]
Nanofiber	poly-N-acetyl glucosamine	polydeoxyribonucleotide	High rate of cell proliferation and angiogenesis.	[63]
Nanofiber	polyethersulfone	henceforth CD34+ cells	The fast diabetic wound healing process	[64]
Nanofiber	PCL	Bixin	Sustained drug release and accelerated wound healing.	[65]
Nanofiber	PLGA	PDGF, vancomycin, and gentamicin	Sustained drug release and accelerated wound healing.	[66]
Nanofiber	PCL	Sodium percarbonate	Superior vascularization.	[67]
Nanofiber	Cellulose acetate	Ag nanoparticles	High antibacterial efficacy and accelerated diabetic wound contraction.	[68]
Nanofiber	PCL	Curcumin	Excellent biocompatibility and increased rate of wound reduction	[69]
Nanofiber	PLGA	Insulin	Good mechanical performance and prolong drug release	[70]
Nanofibers	Chitosan and PVA	ZnO	Excellent antibacterial effects and accelerated diabetic wounds	[71]
Nanofibers	PVP and PCL	Pioglitazone	Non-toxicity and sustained drug release.	[72]
Film	Sodium alginate	Vicenin-2	Faster diabetic wound recovery	[76]
Film	Chitosan	Alcoholic extracts	Excellent biocompatibility	[77]
Film	Chitosan	Fibroblast growth factors	High diabetic wound contraction rate	[78]
Film	Cellulose and PVA	Propolis and vitamin C	High swelling rate, controlled drug release, and accelerated diabetic wound healing	[79]
Film	Fibroin	Aloe gel	Excellent mechanical properties and fibroblast distribution and collagen fiber organization.	[80]
Film	Fibroin and chitosan	ADSCs	Good diabetic wound closure.	[81]
Film	PVA and cellulose	Curcumin	Good antibacterial effects and significantly diabetic wound closure.	[82]
Film	Chitosan	Retinoic acid	Increased wound reduction rate.	[83]
Film	Collagen	Biotinylated GHK peptide	Accelerated wound healing	[84]
Film	PVP and PVA	Sodium fusidate	Excellent mechanical performance	[85]
Film	Cellulose	Selenium	Fast diabetic wound healing rate	[86]
Membrane	PHBV	Cerium Oxide nanoparticles	Significant enhancement in cell infiltration and granulation tissue formation	[88]
Membrane	PVA and PLA	GFs	Excellent cell migration and proliferation	[89]
Membrane	HA	Human keratinocytes	The good clinical wound healing process	[90]
Membrane	PLGA and collagen	Glucophage	The faster wound healing process	[91]
Membrane	PLGA	Metformin	Enhanced the wound healing and re-epithelialization in diabetic rats	[92]
Membrane	PLLA	Dimethyloxalylglycine	Burst drug released followed by sustained drug release.	[93]
Membrane	Cellulose acetate	Sesamol	Improved diabetic wound healing	[94]
Membrane	PLGA and cellulose	Neurotensin	Sustained drug release and faster wound healing process	[95]
Hydrogel	Poly-ε-L-lysine, HA, and pluronic	Adipose mesenchymal stem cells	Increased diabetic wound rate	[100]
Hydrogel	HA and PEG	Stem cell	Good mechanical properties and faster diabetic wound healing.	[101]
Hydrogel	PEG and PVA	Fibroblasts and insulin	Accelerated wound repair	[102]
Hydrogel	HA	Human adipose stem cells	Improved wound closure rate	[103]
Hydrogel	Gelatin	Chemotactic cytokines	Accelerated wound healing	[104]
Hydrogel	Sodium carboxymethylcellulose	*B. orientale*	Fast wound recovery	[105]
Hydrogel	Pluronic F-127	ADSCs	Accelerated wound healing	[106]
Hydrogel	PU	AASCs	Fast diabetic wound	[107]
Hydrogel	Chitosan	Exosomes	Accelerate angiogenesis and wound surface re-epithelialization	[108]
Hydrogel	PPCN	SDF-1	Improved epithelial maturation and granulation tissue production	[109]
Hydrogel	Konjac glucomannan	Avena sativa	Support collagen expression, keratinocyte migration, fibroblast attachment, and proliferation	[110]
Hydrogel	Chitosan	L-glutamic acid	Promotes collagen deposition and accelerates vascularization	[111]
Hydrogel	Gelatin	Curcumin	Good cell migration	[112]
Hydrogel	Chitosan and PEG	Ag nanoparticles	Controlled drug release and diabetic wound stimulation.	[113]
Hydrogel	poly-(polyethyleneglycol citrate-co-N-isopropylacrylamide)	Copper metal-organic framework	Enhanced dermal cell migration and improved wound closure rates	[114]
Hydrogel	PVA	Nitric Oxide	Enhance diabetic wound healing	[115]
Hydrogel	HA	DNA	Enhanced development of granulation tissue	[116]
Hydrogel	poly (γ-glutamic acid) and chitosan	Superoxide dismutase	Good cytocompatibility and accelerated wound healing process	[117]
Hydrogel	Gelatin	Cerium-containing bioactive glass nanoparticles	Good antibacterial effects	[118]
Hydrogel	chitosan-dextran	Ag nanoparticles	Broad-spectrum and long-lasting antibacterial activity	[119]
Foam	PU	RhEGF	Moderate WVTR and good biocompatibility	[122]
Foam	PVA	Gentian violet and methylene blue	High wound reduction rate	[123]
Foam	PU	Ag nanoparticle	Fast wound healing rate	[124]
Foam	PU	Ag nanoparticle	Good antibacterial efficacy	[125]
Foam	PU	Ag	Good diabetic wound closure	[126]
Foam	Silk fibroin	Gastrodia elata and tea tree oil	High porosity and excellent biocompatibility	[127]
Foam	Chitosan	Neurotensin	High wound healing reduction	[128]
Foam	Silicone	Silver	Positive diabetic wound closure and reduction in size	[129]
Wafer	Calcium alginate	Ciprofloxacin	High porosity and burst drug release followed the sustained release with good antibacterial efficacy	[132]
Wafer	Xanthan gum	Silymarin	Good cell migration	[133]
Wafer	Sodium alginate and gelatin	Diosmin nanocrystals	Sustained drug release and well-developed granulation tissue, well-organized dermal layers, complete re-epithelialization, and mature collagen bundles in diabetic wounds.	[134]
Sponges	Chitosan and collagen	Recombinant human acidic fibroblast growth factors	Enhanced diabetic wound healing	[138]
Sponge	HA and chitosan	Ag nanoparticle	Good antibacterial effects and good cytocompatibility	[139]
Sponge	Chitosan	TMC nanoparticles	Faster diabetic wound healing	[140]
Sponge	Chitosan and HA	VEGFs	Burst release of GFs followed by sustained release.	[141]
Sponge	HA and collagen	EGF	Promoted blood vascular formation and granulation tissue development.	[142]
Sponge	Chitosan and silk	GMSC-derived exosomes	Enhanced deposition, re-epithelialization, and remodeling of ECM	[144]
Sponge	Collagen	Gementacin	Good pathogen eradication in diabetic wound	[143]
Sponge	Chitosan and alginate	Curcumin and honey	Sustained drug release and faster wound healing	[145]
Sponge	Chitosan and collagen	Thymosin beta 4	Enhanced diabetic cutaneous wound healing	[146]
Bandages	Chitosan	ZnO nanoparticles	Good cytocompatibility and antibacterial effects.	[147]
Bandage	Chitin	ZnO nanoparticles	excellent antibacterial activity and high cell adhesion and migration	[149]
Bandage	Sodium alginate	EGF and curcumin	Non-toxicity and good biocompatibility	[148]
Bandage	Chitosan	Fluconazole and ciprofloxacin	High porosity and sustained drug release with good antimicrobial effects	[150]

**Table 2:** Comparison of various types of wound dressings.

Types of Wound Dressings	Advantages	Disadvantages	Highlights
Nanofibers	They possess a structure that mimics ECM, making them suitable for skin wound healing and regeneration. They are frequently formulated using efficient and easily employed electrospinning techniques.	It is not easy to produce nanofibers less than 10 nm in diameter.	The SEM micrographs of nanofibers loaded with bioactive agents display bead-free morphology that mimics ECM, making these wound dressings appropriate for providing an environment for cell proliferation and adhesion to accelerate the diabetic wound healing process.
Films and Membranes	These wound dressings are transparent, showing that the wound healing process can be observed without removing them. They also display good mechanical performance.	They are not suitable for exuding wounds due to their inability to absorb a large volume of biological fluids.	The mechanical properties of films and membranes were like those of human skin, making them skin compatible and easily handled during diabetic wound management.
Hydrogels	They are used as potential drug delivery systems in wound dressing applications and display other interesting properties such as high porosity, high swelling capacity, excellent biocompatibility, etc.	The biopolymer hydrogel dressings demonstrate poor mechanical performance that makes them not compatible with the human skin.	The drug release profiles were controlled release of bioactive agents from the polymeric hydrogels, resulting in an improved wound healing process. The high porosity of the hydrogels led to good swelling capability.
Foams and Wafers	These wound dressings exhibit high porosity that could provide cell growth and adhesion to accelerate the wound healing process.	They are not suitable for dry wounds.	The WVTR experiments of foams and wafers loaded with drugs exhibited moderate WTVR that can provide appropriate moisture to accelerate the healing of diabetic wounds.
Sponges and Bandages	These wound dressings are also displayed high porosity that could offer suitable gaseous permeation, superior cell proliferation, migration, and attachment for the accelerated wound healing process.	The very high porosity of the polymeric sponges or bandages can result in high uptake of wound exudate and high WVTR that may cause wound dehydration.	Polymeric sponges and bandages were mostly loaded with antibacterial agents for diabetic wound treatment, and they exhibited excellent antibacterial activity, demonstrating that these dressings are potential candidates for the management of infected diabetic wounds

## Data Availability

Not applicable.
